# Titanium Dioxide Grafted on Graphene Oxide: Hybrid Nanofiller for Effective and Low-Cost Proton Exchange Membranes

**DOI:** 10.3390/nano10081572

**Published:** 2020-08-10

**Authors:** Cataldo Simari, Ernestino Lufrano, Nicolas Godbert, Dimitrios Gournis, Luigi Coppola, Isabella Nicotera

**Affiliations:** 1 Department of Chemistry and Chemical Technologies, University of Calabria, 87036 Rende (CS), Italy; cataldo.simari@unical.it (C.S.); ernestino.lufrano@unical.it (E.L.); nicolas.godbert@unical.it (N.G.); lg.coppola@unical.it (L.C.); 2 Department of Material Science and Engineering, University of Ioannina, 45110 Ioannina, Greece; dgourni@cc.uoi.gr

**Keywords:** graphene oxide, nanocomposite membranes, PEMFCs, proton transport, sulfonated polysulfone

## Abstract

A nanostructured hybrid material consisting of TiO_2_ nanoparticles grown and stabilized on graphene oxide (GO) platelets, was synthesized and tested as nanofiller in a polymeric matrix of sulfonated polysulfone (sPSU) for the preparation of new and low-cost nanocomposite electrolytes for proton exchange membrane fuel cell (PEMFC) applications. GO-TiO_2_ hybrid material combines the nanoscale structure, large interfacial area, and mechanical features of a 2D, layered material, and the hygroscopicity properties of ceramic oxides, able to maintain a suitable hydration of the membrane under harsh fuel cell operative conditions. GO-TiO_2_ was synthetized through a new, simple, one-pot hydrothermal procedure, while nanocomposite membranes were prepared by casting using different filler loadings. Both material and membranes were investigated by a combination of XRD, Raman, FTIR, thermo-mechanical analysis (TGA and Dynamic Mechanical Analysis) and SEM microscopy, while extensive studies on the proton transport properties were carried out by Electrochemical Impedance Spectroscopy (EIS) measurements and pulse field gradient (PFG) NMR spectroscopy. The addition of GO-TiO_2_ to the sPSU produced a highly stable network, with an increasing of the storage modulus three-fold higher than the filler-free sPSU membrane. Moreover, the composite membrane with 3 wt.% of filler content demonstrated very high water-retention capacity at high temperatures as well as a remarkable proton mobility, especially in very low relative humidity conditions, marking a step ahead of the state of the art in PEMs. This suggests that an architecture between polymer and filler was created with interconnected routes for an efficient proton transport.

## 1. Introduction

Proton exchange membrane fuel cells (PEMFCs) are recognized worldwide as one of the most promising clean energy conversion technologies [1,2]. Owing to their high chemical-to-electrical energy conversion efficiency, and due to their zero-CO_2_ emission (when H_2_ is used as fuel), PEMFCs are targeted as an energy source for electric vehicles, although they are also proposed for a wide range of applications, from portable to automotive and stationary. However, there are still several issues to be addressed for a large scale-up of this technology. Most of them are related to the intrinsic properties of the electrolyte membrane (proton exchange membrane-PEM), in particular its ability to maintain adequate proton conductivity even under drastic operating conditions, i.e., working temperatures above 100 °C (from the current 70–90 °C) and low humidification. Indeed, increasing the temperature (the DOE, Department of Energy USA, fixed the temperature target at 120 °C), the cell performance improves because (1) higher electrode reaction rates take place, (2) simplified and more effective water management in the cell occurs, (3) enhanced CO tolerance by the electrocatalyst (Pt) is induced, and (4) faster heat rejection rates and better systems integration are observed [3]. The state-of-the-art PEM is based on Nafion^®^, a perfluorosulfonic acid ionomer (PFSA), developed in the 1960s by Dupont, which is still, nowadays, the most used membrane both in fuel cell research and industry, since it combines high proton conductivity, excellent chemical stability, and good mechanical resistance. However, under the operative conditions of high temperature and low humidification (required in several applications such as automotive), Nafion membranes easily loose water, so that the proton conductivity decreases of several orders of magnitude with respect to the fully hydrated membrane. Furthermore, its high environmental impact, due to its content in fluorine atoms, and its elevated production costs are both issues that are driving the research toward alternative proton conductive ionomers [4]. In this regard, great efforts have been devoted to the development of non-fluorinated polymers (n-FPs) [5]. A large variety of aromatic thermoplastics ionomers, such as sulfonated polyether ether ketone [6,7], sulfonated polyphenylsulfone [8], phosphoric acid doped polybenzimidazole [9,10], sulfonated polyether sulfone [11,12,13], sulfonated polysulfone [14,15], and sulfonated polyimides [16], is being actively investigated, showing high thermo-oxidative, chemical, and mechanical stabilities. Additionally, several approaches have been developed to improve the performance of n-FPs, including the modification of the ionomer backbone with specific functional groups, the production of polymer blends and the preparation of composites’ membranes by introduction of proper hygroscopic nanoadditives [17,18]. Accordingly, most of the attention has been directed toward the preparation of organic-inorganic composite membranes because they offer the possibility to effectively combine both the thermo-mechanical stability of the polymer backbone and the chemical reactivity of the filler [19]. For instance, the incorporation of SiO_2_, ZrO_2_, TiO_2_, zeolites, zirconium hydrogen phosphonates, and Layered Doubled Hydroxide (LDH) materials has been demonstrated to remarkably improve the physico-chemical and mechanical properties of the resulting composite membranes, as well as the electrochemical behavior since the high acid surface area and hydrophilicity of such nanofillers usually favor the proton transport [20,21,22,23].

Among the nanostructured materials, graphene oxide (GO), produced by oxidation of graphite, has been considered attractive for many applications owing to its fascinating physical and chemical features [24]. While still preserving the typical layered structure of the parent graphite, which provides a large surface area, GO exhibits also electronic insulating property, high dispersive capacity, and remarkable proton conduction features [25]. These features endow GO with great potential as a nanofiller in PEMs for PEMFCs applications. Indeed, GO has been successfully incorporated into Nafion, either alone or coupled with other active components with the main objective to boost proton transport [26,27,28]. However, in order to really be efficient, proper surface chemical functionalization must be performed to enhance the number of acid/hydrophilic groups, thus providing additional continuous pathways for facile proton transport [29]. In fact, it has been largely reported that the incorporation of sulfonated graphene oxide remarkably improves the transport and mechanical features of Nafion, sulfonated polyimide, and polybenzimidazole [30,31,32]. Similar outcomes were also exhibited after introduction of both phosphorylated graphene oxide [33] and polydopamine-modified GO [34] nanofillers. 

In this work, a hybrid nanoadditive, which combines the proton conduction characteristic of GO with the water-holding ability of inorganic oxides, such as TiO_2,_ was proposed. Titanium oxide nanoparticles are high surface area hygroscopic ceramic oxides, which demonstrate good compatibility with organic solvents and polymers as well as displaying good hydrophilicity, mechanical, and thermal stabilities [35]. However, in the preparation of composite membranes, one of the most important and challenging aspects is to obtain an effective nanodispersion of the filler within the polymeric matrix, avoiding the formation of micrometric agglomerates, usually observed with ceramic oxides’ nanoparticles [36]. Therefore, in this work, titania nanoparticles were dispersed and stabilized on graphene platelets by chemical grafting in order to allow homogeneous dispersion and create a nanoadditive able to maintain high proton conductivity even under elevated-temperature and low-humidity conditions. 

To this purpose, titania-decorated graphene oxide (GO-TiO_2_) was successfully synthesized via a one-pot thermal hydrolysis of titanium(IV) butoxide used as TiO_2_ precursor in a GO highly dispersed suspension. 

Usually, graphene/TiO_2_ nanomaterials are synthesized under extreme or special conditions, i.e., high-pressure atmosphere [37], high temperature in autoclave [38], or UV irradiation [39]. Although successful, these methods are probably not commercially viable since they often require multistep or expensive processes. Herein, we report a simple one-pot hydrothermal procedure, conducted at intermediate temperature (60 °C). This makes our procedure more attractive and suitable to industrial-scale process.

Subsequently, GO-TiO_2_ nanohybrid was dispersed in sulfonated polysulfone (sPSU) for the preparation of low-cost and more eco-friendly proton exchange membrane with respect to the more commonly studied and used Nafion. Polysulfone was selected as a polymeric matrix due to its numerous interesting characteristics ranging from low cost, large availability, low environmental impact, and good film-forming capacity, to its considerable thermo-mechanical strength. For applications in PEMFCs, the transport and structural features of polysulfone can be easily tuned through the degree of sulfonate groups introduced, which can be controlled by choosing the sulfonation procedure. In this report, the sPSU was successfully synthesized according to the procedure proposed by Lufrano et al., which involves the use of trimethylsilylchlorosulfonate as sulfonating agent and allowed us to suitably and easily calibrate degree of sulfonation through reaction times [15,40]. The final product had an ion exchange capacity (IEC) of 1.36 meq g^−1^, selected as the compromise value between the structural stability of the macromolecule and the appropriate proton transport properties.

The sPSU_GO-TiO_2_ nanocomposite membranes, at various additive loadings, were prepared by the solution-casting procedure adequately tuned to ensure a homogeneous dispersion. As of now, to our best knowledge, GO–TiO_2_ hybrid material has not been investigated in nanocomposite electrolytes for PEMFC applications. Both the hybrid nanofiller and nanocomposite membranes were characterized by a combination of powder X-ray diffraction (XRD), Raman and Fourier-transform infrared (FTIR) spectroscopies, and thermogravimetric analysis (TGA). The morphological features were investigated by scanning electron microscopy, while the mechanical properties of the membranes were tested by dynamic mechanical analysis (DMA) in a wide temperature range. A thorough investigation of the proton transport properties and of water molecular dynamics inside the hydrophilic clusters of the nanocomposite membranes was conducted by the pulse field gradient (PFG) NMR spectroscopy. Finally, ac impedance spectroscopy was carried out to clarify the influence of the GO-TiO_2_ nanohybrid on both the proton conductivity and hydrolytic stability of the resulting PEMs.

## 2. Materials and Methods 

### 2.1. Materials and Chemicals

Graphite powder (powder < 20 mm), sulfuric acid (95–97 wt.%), nitric acid (65 wt.%), potassium chlorate powder (purum, >98.0%), titanium(IV) butoxide (reagent grade, 97%), isopropyl alcohol (ACS reagent, ≥99.5%) and N,N-dimethylacetamide (anhydrous, 99.8%) were all purchased from Sigma-Aldrich and used as received. The sPSU with ion exchange capacity of 1.36 meq g^−1^ was synthesized as reported elsewhere [15].

### 2.2. Synthesis of Graphene Oxide

Graphene oxide (GO) was prepared according to the following procedure [41,42]: 10 g of graphite powder and 200 g of potassium chlorate powder were slowly added to a mixture of sulfuric acid and nitric acid (400:200 mL, respectively). The reaction was conducted in an ice bath under vigorous magnetic stirring for 18 h and thus quenched by pouring the mixture into distilled water. The final GO product was centrifuged, washed several times with distilled water until the pH = 6.0, and was finally dried at room temperature.

### 2.3. Functionalization of GO with TiO_2_ (GO-TiO_2_)

**Scheme 1:**
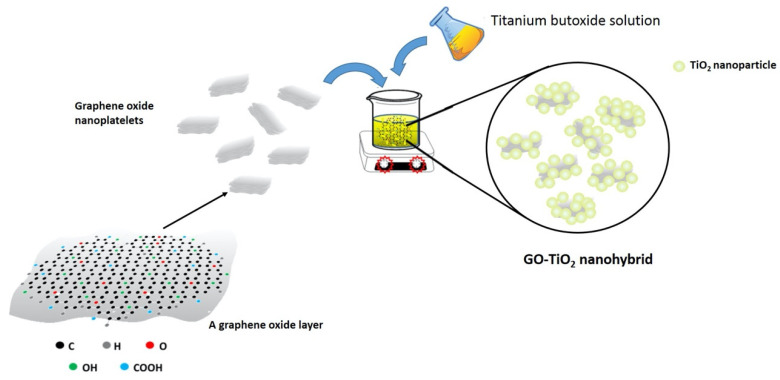
Synthesis route of the GO-TiO_2_ nanohybrid material.

GO-TiO_2_ was synthesized via thermal hydrolysis of titanium(IV) butoxide in a GO suspension, as illustrated in Scheme 1. In a two-neck, round-bottom flask, titanium(IV) butoxide was added to a mixture of water, isopropyl alcohol, and nitric acid. The solution was heated to 60 °C in an oil bath and stirred for 1 h to produce a Titanium(IV) ionic (Ti^4+^) solution. Subsequetly, a homogenous aqueous dispersion of exfoliated GO was slowly added dropwise and the resulting mixture was stirred at 60 °C for 18 h. The titanium butoxide/GO mass ratio was 14:1. The dispersion was then centrifuged at 15,000 r min^−1^ (*ca.* 26,000 g) for 10 min and the deposited solid was repeatedly washed with deionized water and ethanol. Finally, the GO-TiO_2_ sample was vacuum-dried at 60 °C overnight prior to characterization. The GO-TiO_2_ nanohybrid material was obtained as a black, fine powder.

### 2.4. Preparation of the Nanocomposite Membranes

The sPSU-based composite membranes at three different filler loadings, i.e., containing 2, 3, and 5 wt.% of GO-TiO_2_, were prepared by solution-casting procedure. Typically, 150 mg of sPSU were dissolved in 10 mL of N,N-dimethylacetamide at room temperature to form a clear solution. Then, the appropriate amount of the GO-TiO_2_ nanohybrid was directly added in the sPSU-DMAc polymer solution, ultrasonicated for 1 day, and stirred for another day at room temperature until a macroscopically homogeneous dispersion was obtained. The mixture was cast on a petri dish at 80 °C and left to dry. A pure sPSU membrane was also prepared through identical procedure. Before any characterization, all membranes were pretreated by immersing in 1 M H_2_SO_4_ solution at 60 °C for 15 h and finally rinsed in deionized water to remove any residual trace of acid. The dry thickness of the membranes was 40 ± 10 μm.

### 2.5. Characterization of GO, GO-TiO_2,_ and Composite Membranes

X-ray diffraction (XRD) measurements were performed using the Cu-Kα radiation of a Bruker AXS Diffractometer/Reflectometer (D8) (Bruker, Karlsruhe, Germany) equipped with a Dynamic Scintillation Detector, NaI, and with a Gobel mirror. The experimental tests were made in transmission, and the powder samples carefully were put in glass capillaries (*ϕ* = 1 mm, Hilgen-berg GmbH, Karlsruhe, Germany) and then a sample holder [43]. Spectra were collected at room temperature in the 2*θ* range from 5° to 40°, in steps of 0.03° and the counting time was 1 s/step.

The FTIR spectra were recorded in the range of 400–3600 cm^−1^ using a Perkin-Elmer Spectrum GX instrument with KBr pellets. Each spectrum was the average of 64 scans collected at 2 cm^−1^ resolution, and these were averaged to improve the signal-to-noise-ratio.

The Raman spectra were recorded with a Jobin Yvon micro-Raman LABRAM apparatus (HORIBA Ltd., Kyoto, Japan). The 632.8-nm wavelength of a He–Ne laser was used as an excitation source. An objective 50× Olympus lens with a focal length of 15 mm was used. The resulting power was about 5 mW, focused on a spot of about 5 μm of diameter. The interaction at molecular levels of the organo-modified derivatives can be investigated by studying the changes of Raman spectra. To have a complete view of such phenomena, the Raman spectra were collected in the range of 200 to 3900 cm^−1^.

Thermogravimetric analyses (TGA) were carried out in a TG-7 instrument from Perkin-Elmer. The samples were heated from 50 to 900 °C under flowing nitrogen, at a heating rate of 10 °C min^−1^. Prior to the measurements, samples were dried in an oven at 60 °C for 24 h.

SEM morphologic images and energy dispersive X-ray spectroscopy (EDX) were acquired on a Phenom ProX scanning electron microscope (Thermo Fisher Scientific Inc., Waltham, MA, USA) equipped with a backscattered electron detector. Samples were placed on carbon-conductive, double-coated tabs and were observed and analyzed without coating. Membranes’ sections were performed on samples that were cut under freezing condition after immersion in a nitrogen liquid bath.

Conventional titration method was used in this work to determine the ion exchange capacity (IEC in milliequivalents per g of dry polymer) of the functionalized sPSU-based membranes [12]. The procedure was determined by potentiometric acid-base titration. After the exchange with H^+^ ions, the membranes were washed in deionized water and dried over P_2_O_5_ for 24 h. Subsequently the samples were weighed (*W_dry_*) and immersed in a solution of 0.5 M NaCl solution for 48 h at 60 °C. The resulting solution was then back-titrated with a solution of NaOH 0.1 M using phenolphthalein as an indicator. The IEC value is calculated by the following Equation (1).

(1) $$IEC=\frac{V_{NaOH}\;\cdot \;C_{NaOH}}{W_{dry}};\;[\mathrm{meq}\;g^{-1}]$$

Water uptake was measured by immersing the dried membranes (*W_dry_*) in deionized water at room temperature for 24 h. The membrane was then quickly dried with tissue paper to remove surface water droplets and weighed (*W_wet_*). The water uptake was calculated by Equation (2) and reported as an average of three independent measurements.

(2) $$w.u=\frac{W_{wet}\;-\;W_{dry}}{W_{dry}}\cdot 100;\;[\%]$$

NMR measurements were performed on a Bruker AVANCE 300 wide-bore spectrometer working at 300 MHz on ^1^H with a Diff30 Z-diffusion 30 G/cm/A multinuclear probe with substitutable RF inserts. The self-diffusion coefficients (*D*) of water confined in the membranes have were measured by pulsed field gradient stimulated-echo (PFG-STE) sequence [44], by using the following experimental parameters: Pulse gradient length (*δ*) 1 ms, diffusion delay time (Δ) 10 ms, and the gradient amplitude (*g*) varied from 100 to 900 G/cm. The number of scans was 8. Based on the very low standard deviation of the fitting curve and repeatability of the measurements, the uncertainties in D values were calculated to be circa 3%. The NMR samples were prepared according to the procedure reported elsewhere [45] and the measurements were conducted by increasing the temperature from 20 °C to 130 °C, every 20 °C, by equilibrating the sample for about 15 min at each temperature.

Dynamic mechanical analysis (DMA) was conducted by Metravib DMA/25 analyzer equipped with a shear jaw for films’ clamping. A dynamic stress of amplitude 10^−4^ at 1 Hz was applied on a rectangular-shaped sample, in the temperature range 25–180 °C, with a heating rate of 2 °C min^−1^.

Proton conductivity (*σ*) was measured by a homemade, two-electrodes cell in a through-plane configuration. A circular-shaped membrane was sandwiched between two sheets of conductive carbon papers and placed between two graphite-blocking electrodes. The cell was placed between the anode and cathode flow field of a fuel cell test hardware (850C, Scribner Associates Inc., Southern Pines, North Carolina, NC, USA). The AC impedance response of the cell was recorded using a PGSTAT 30 potentiostat/galvanostat (Methrom Autolab, Utrecht, Netherlands) equipped with a Frequency Response Analyser (FRA) module. Voltage amplitude was 10 mV and the frequency range was 1 Hz–1 MHz. The obtained impedance data were analyzed by Metrohm Autolab NOVA software and the electrolyte resistance (R_el_) was determined from the high-frequency intersection with the real axis in Nyquist plot. Measurements were performed in the temperature range from 20 °C up to 130 °C at the relative humidity (RH) of 90%. Proton conductivity (*σ*) of the electrolyte membrane was calculated according to Equation (3).

(3) $$\sigma =\frac{L}{A\;\cdot \;R_{el}};\;[S\;{\mathrm{cm}}^{-1}]$$

where *L* is the distance between the electrodes, *R* is the resistance, and *A* is the active area.

Finally, the hydrolytic stability of the membranes was assessed in terms of relative variation of *σ* during long-time operation. The membranes were kept at 80 °C under 100% RH and the proton conductivity was measured at each interval of time (*σ_t_*) for 140 h. For each measurement, the relative variation in proton conductivity (Rv_*σ*_) was calculated according to Equation (4):

(4) $${\mathrm{Rv}}_{\sigma }=\left(1-\frac{\sigma_{0}-{\;\sigma }_{t}}{\sigma_{0}}\right)\cdot 100;\;[\%]$$

where *σ*_0_ is the initial proton conductivity.

## 3. Results and Discussion

### 3.1. Graphene Oxide (GO) and GO-TiO_2_ Hybrid Nanomaterial

**Figure 1:**
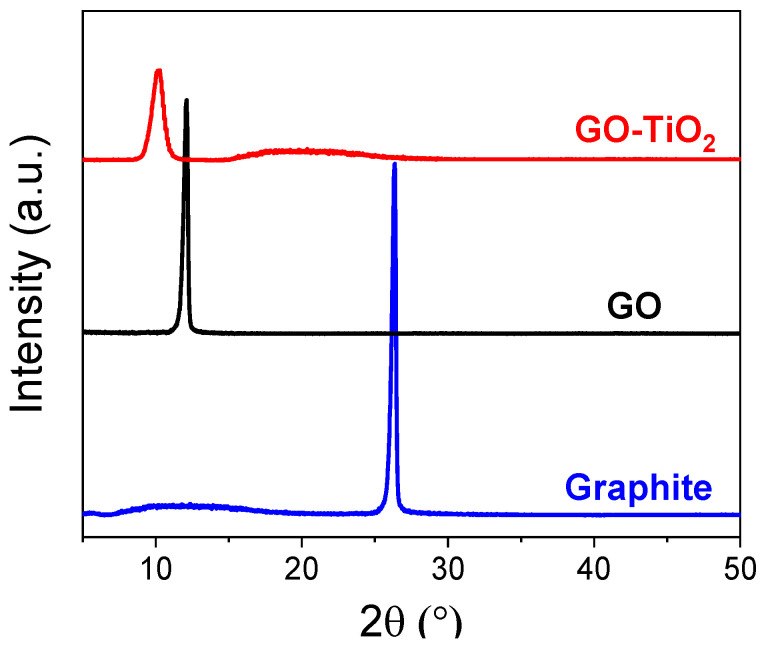
XRD patterns of graphene, GO, and GO-TiO_2_ nanostructured materials.

XRD patterns of graphite, GO, and GO-TiO_2_ materials are shown in Figure 1. Pristine graphite exhibited a characteristic diffraction peak at 2*θ* of 26.3°, corresponding to a basal d_002_ = 0.34 nm [46]. Graphene oxide, instead, showed a strong basal reflection peak at 12.0° with a d_001_ = 0.73 nm. Such increasing of the d-spacing is clearly amenable to the introduction of oxygen-containing functional groups between the layers, confirming the successful oxidation of graphite to graphene oxide [29]. An additional increase in d-spacing (d_001_ = 0.86 nm) was observed for GO-TiO_2_ hybrid material, with a broader and shifted basal reflection peak at 2*θ* = 9.97°, corresponding to an intersheet separation of Δ = 0.86 − 0.61 = 0.25 nm, where 0.61 nm is the thickness of the GO monolayer [31]. This slight increase of the interlayer distance and the broadening of the associated reflection peak can be justified, taking into account two concomitant factors: (1) The use of nitric acid to promote the hydrolysis of titanium(IV) butoxide introduces more oxygen-containing functional groups onto the GO nanoplatelets resulting, in turn, in a higher separation of the graphene-oxide layers [47,48] and (2) the reaction solution, inducing the exfoliation of the GO sheets, reduces the size of sheet stack (the thinner it is, the bigger is the reflection line broadening); therefore, a more relaxed stack results in a larger distance between individual GO sheets. With Scherrer’s equation, we can roughly estimate the stack thickness and the average number of layers: For GO it is 27 nm with about 37 layers, which is reduced to about 9 nm with 10 layers for GO-TiO_2_.

In both cases, however, the presence of TiO_2_ nanoparticles grown onto the GO surface platelets prevented the restacking and the GO remained mostly exfoliated. 

Finally, no diffraction peaks attributable to reflections from anatase or rutile crystal phases were observed [49], suggesting the complete amorphous nature of the TiO_2_ nanoparticles in the as-prepared nanohybrid. Indeed, the XRD pattern of the GO-TiO_2_ nanohybrid displays, together with the broader and shifted basal reflection peak with respect to the starting GO, a halo centered at 2*θ* = 18°, evidence of the amorphous nature of the synthesized TiO_2_ (Figure 1).

**Figure 2:**
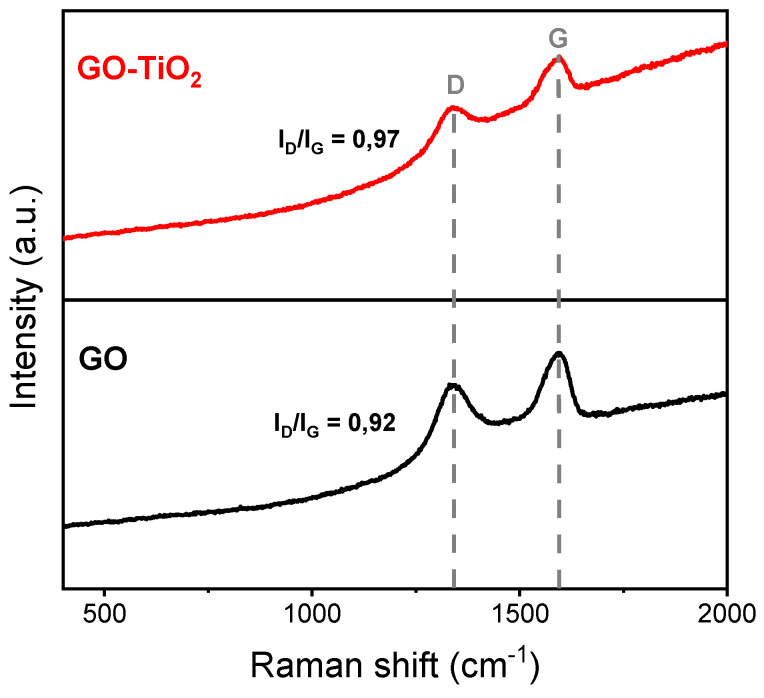
Raman spectra of GO and titania-functionalized GO.

The crystalline structure of GO and GO-TiO_2_ were further investigated by Raman analysis, the spectra of which are shown in Figure 2. The characteristic G- and D-bands of graphene oxide are observed at ~1607 and ~1352 cm^−1^, respectively; the former related to the in-plane vibrations of sp^2^-bonded carbon atoms, the second to the presence of sp^3^ defect sites associated with vacancies and grain boundaries [50]. The peak intensity ratio (*I*_D_/*I*_G_), which gives a measure of the structural defects in the graphite layer, was found to be 0.92 for the GO. Raman spectrum of the GO-TiO_2_ nanohybrid still displayed the two major characteristic peaks of graphene oxide and no additional crystalline bands were observed. This corroborates the amorphous nature of titanium dioxide rooted on the GO platelets. Additionally, the *I*_D_/*I*_G_ ratio increased to 0.97, proving the partial reduction of GO following the hydrothermal process. Therefore, we can infer that the hybrid material consists of TiO_2_ particles decorating the graphene oxide nanosheets [51].

**Figure 3:**
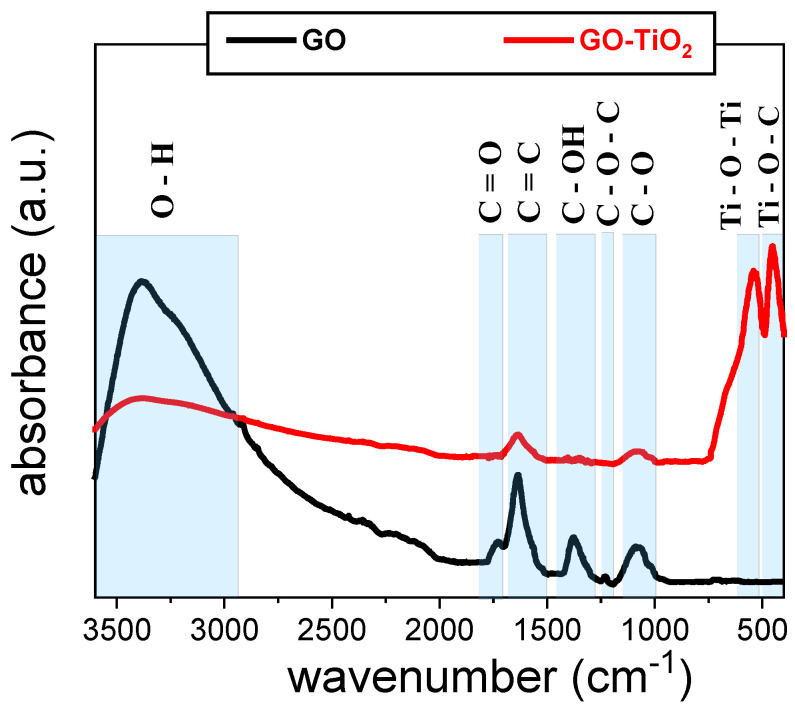
FTIR spectra of GO and GO-TiO_2_ nanomaterials.

FTIR spectra of GO and GO-TiO_2_ nanohybrid in the wavenumber range of 400–3750 cm^−1^ are reported in Figure 3. Graphene oxide exhibits the typical absorption bands of oxygen-containing functional groups: The apparent bands at 1070, 1240, 1384, and 1629 cm^−1^ are assigned to the characteristic vibrations of C–O, C–O–C (epoxy), C–OH (alkoxy), and C=C (aromatics) bonds, respectively. The stretching vibration of C=O (carbonyl) and O–H (hydroxyl) generates the other two absorption bands at 1745 and 3380 cm^−1^, respectively. These bands prove the effective synthesis of GO [25]. All signals strongly decrease in intensity in the GO-TiO_2_ material, suggesting the oxygen-containing functional groups are directly involved in the hydrolysis of the titania precursor [52]. Furthermore, two well-defined absorption bands also appear in the fingerprint region. This clearly originates from both the Ti–O–C stretching and the Ti–O–Ti vibration, generally occurring at 460 cm^−1^ and 530 cm^−1^, respectively [53]. Such features definitively confirm the TiO_2_ particles are chemically bonded to GO nanoplatelets, thus clearly proving the successful functionalization of graphene oxide embedded with TiO_2_ nanoparticles. Further undeniable evidence will be brought by SEM images and EDX analysis, reported below in the corresponding section.

**Figure 4:**
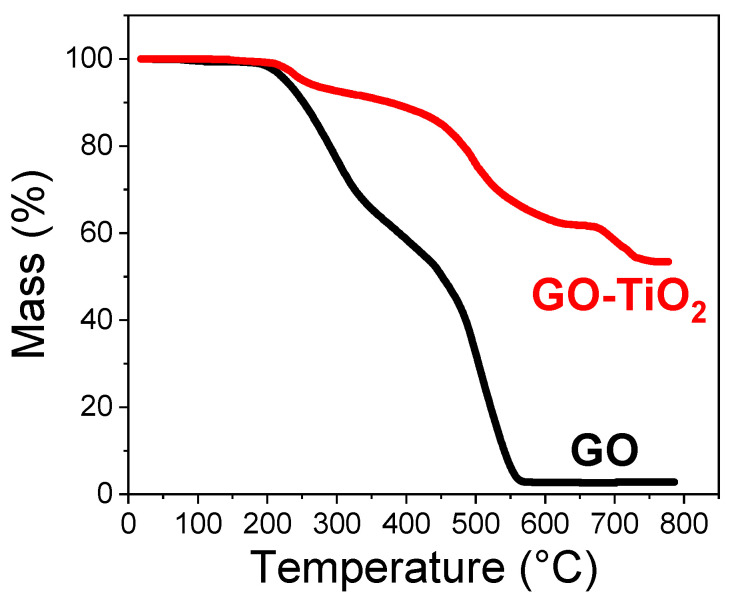
Thermogravimetric analysis (TGA) of GO and GO-TiO_2_ materials.

The TGA curves in Figure 4 show the weight loss of graphene oxide and its TiO_2_ derivative. The degradation of graphene oxide starts at almost 200 °C following the decomposition of oxygen-containing functional groups, whereas its honeycomb structure deteriorates above 400 °C. For the GO-TiO_2,_ a third weight loss was also observed at *ca.* 650 °C, likely due to the phase conversion from amorphous TiO_2_ to anatase. Noteworthy, the TGA results allow us to estimate a TiO_2_ content of 53.4% in the nanohybrid material.

### 3.2. The sPSU-Based Nanocomposite Membranes

**Figure 5:**
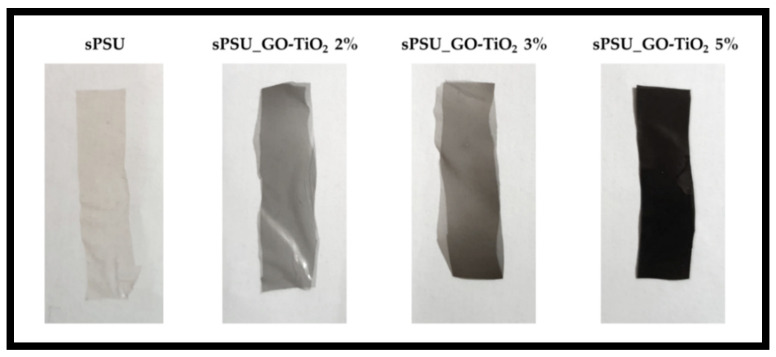
Photographs of sPSU and sPSU_GO-TiO_2_ nanocomposite membranes.

The synthetized GO-TiO_2_ nanohybrid material was used as nanoadditive for the preparation, via simple solution casting method, of sPSU-based nanocomposite membranes at various filler loadings (2, 3, and 5 wt.%, in respect to the polymer). As illustrated in Figure 5, filler-free sPSU membrane is completely transparent, whereas the color of the membranes changes from light gray to black (the color of GO-TiO_2_ powder) as the filler loading increases and, in any case, they appear macroscopically homogeneous, smooth, and without any cracks or defects.

**Figure 6:**
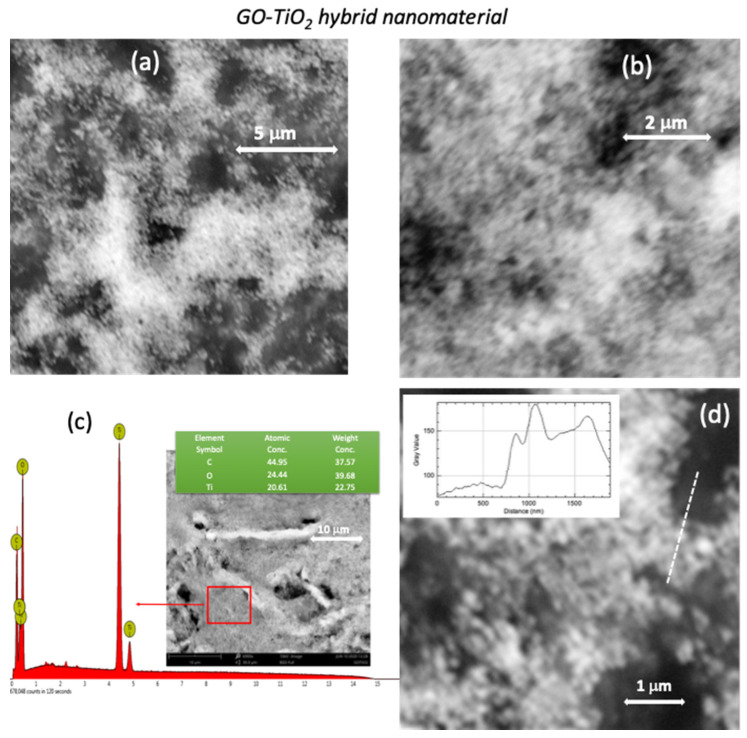

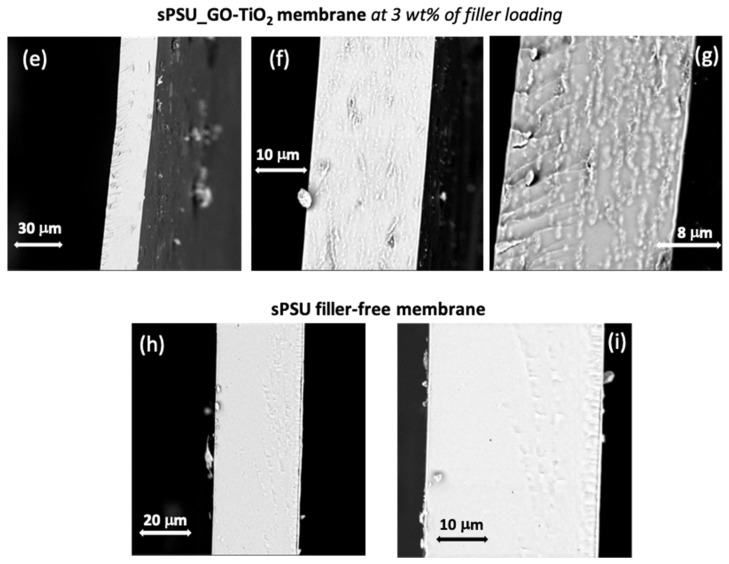
(**a**–**d**) SEM images at various magnifications of GO-TiO_2_ hybrid material and EDX spectrum, (**e**–**g**) cross-section images of sPSU_GO-TiO_2_ composite membrane, (**h**,**i**) cross-section images of filler-free sPSU membrane.

Morphological analyses of the synthetized hybrid GO-TiO_2_ material and membranes were performed by scanning electron microscopy (SEM) and are reported in Figure 6. The Figure 6a shows the nanoparticles of titania dispersed on the graphene platelets. Note that the GO nanoplatelets are no longer visible, due to their complete coverage by TiO_2_ nanoparticles. However, the composition of this material is confirmed by EDX analysis and is shown in the Figure 6b, which clearly reveals the presence of the C, O, and Ti elements only, accordingly with the TiO_2_ particles successfully grown onto the GO platelets’ surface. Higher magnifications images are also shown in Figure 6c,d. The last one reports, in the inset, the intensity profile along the white line showing the typical dimension of the titania particles that range from *ca.* 50 to 150 nm. Noteworthy, the TiO_2_ particles decorating the platelets’ surface should interfere with the restacking and, thus, favor the homogeneous dispersion of the GO-TiO_2_ material in the polymer matrix. Note that some “blur” effect observed mainly on Figure 6b,d at high magnification is only due to the small size of the TiO_2_ nanoparticles (less than 150 nm).

Cross-section images of the sPSU_GO-TiO_2_ and filler-free sPSU membranes at various magnifications are reported in Figure 6e–i, respectively. Both membranes show a dense, compact morphology, without pores or holes. However, while the filler-free sPSU section appears completely smooth and plain, the GO-TiO_2_-based nanoparticles are well visible throughout the whole membrane’s thickness. Furthermore, at higher magnifications, Figure 6f,g, it can clearly be seen that their distribution is characterized by a certain directionality, roughly parallel to the surface. This is definitely related to the lamellar structure of GO, which induces such orientations’ planes, and the TiO_2_ decoration emerges again clearly from these images as white spots.

**Figure 7:**
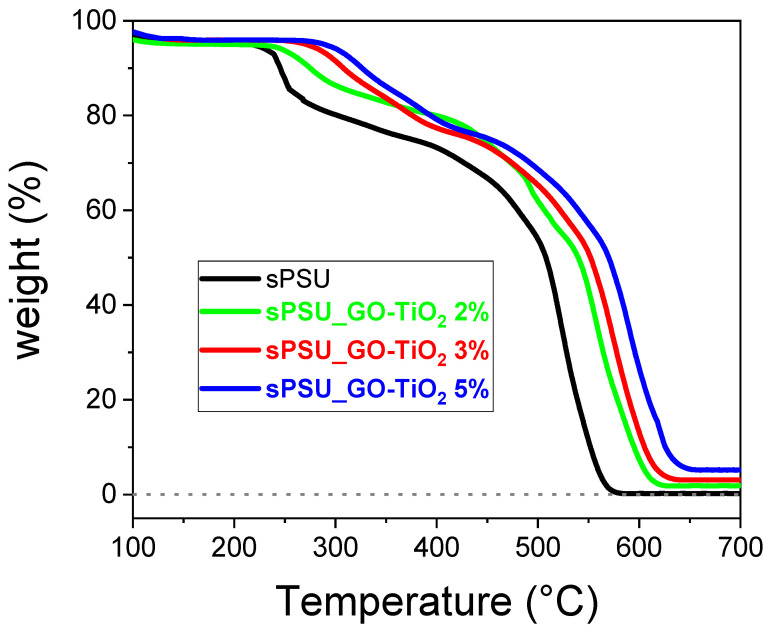
TGA curves of sPSU and sPSU_GO-TiO_2_ composite membranes.

As the core element in the fuel cell, the proton exchange membrane should guarantee excellent thermal and mechanical resistance during the long-time operation. The thermal properties of the membranes were investigated by TGA (Figure 7): Two main degradation weight-loss stages are clearly detectable, the first one between 240 °C and 350 °C, which can be attributed to the pyrolysis of the sulfonic acid groups, and the second stage at about 400 °C, assigned to the degradation of the polymer backbone. In comparison to the filler-free membrane, in the nanocomposites both thermal degradation steps are significantly shifted upward, indicating higher thermal stability than the pristine sPSU. This result suggests a strong interaction between the GO-TiO_2_ hybrid material and the polymer, while the high alteration of the TGA signal upon addition of the filler typically implies that it is homogeneously dispersed in the sPSU matrix [54,55].

**Figure 8:**
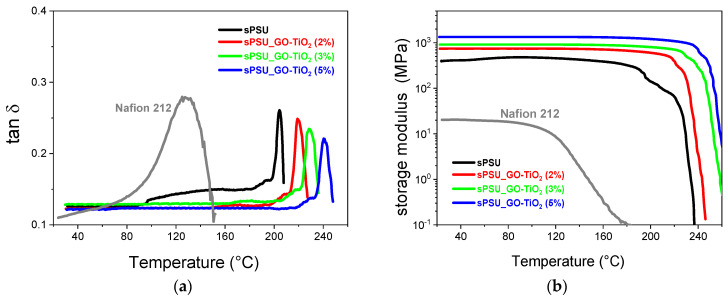
Tan δ (**a**) and storage modulus E’ (**b**) versus temperature of pristine sPSU and nanocomposite membranes containing GO-TiO_2_. Commercial Nafion 212 membrane is also reported for comparison.

The trends observed above are further supported by DMA data. Figure 8a shows the temperature evolution of the tan δ for all the investigated membranes, providing a clear picture of the relationship between the filler content and the thermal stability of the resulting PEM. For comparison, the thermo-mechanical features of the commercial Nafion 212 membrane have also been plotted. The glass transition temperature of the hybrid composite membranes progressively shift toward higher temperature with the increase of the filler loading: From the initial value of about 200 °C of the filler-free sPSU membrane, up to 240 °C for the nanocomposite with 5 wt.% of filler. Compared to Nafion 212, which displays the typical T_g_ at ~125 °C, the thermo-mechanical stability window for these hybrid membranes extends significantly. Moreover, the presence of the GO-TiO_2_ nanohybrid produces a significant increasing of the storage modulus (E’) of the composite membranes (Figure 8b). The highest E’ value is achieved by the sPSU_GO-TiO_2_ (5%) composite film (~1330 MPa), which is about three-fold higher than the sPSU pristine membrane (~400 MPa) and even almost two order of magnitude higher than the Nafion benchmark (19 MPa). Such remarkable result is likely amenable to both the peculiar architecture of GO-TiO_2_ (which combines the mechanical features of 2D, layered materials together with the nanodispersion of the titanium dioxide) and the high chemical affinity between polymer and hybrid material which produces a highly stable network [56,57]. Definitely, the sPSU_GO-TiO_2_ nanocomposites ensure a significant step forward with respect to the state-of-the-art Nafion, since they can effectively withstand higher working temperatures and more severe mechanical stress.

Table 1 reports the ion exchange capacity (IEC) and the water uptake (WU) as a function of the filler content. While the IEC relates to the number of H^+^-transporting functional groups in the membrane, the water uptake is also a crucial factor since it directly affects the proton conductivity and the dimensional stability of the PEM [58]. The presence of the filler produces a sensible increasing of both IEC and WU of the membranes, due to the hygroscope and proton conductor nature of the GO-TiO_2_ nanoplatelets. The maximum values are reached with 3 wt.% of filler, which then goes down with an ulterior load, probably due to some degree of particle agglomeration.

**Figure 9:**
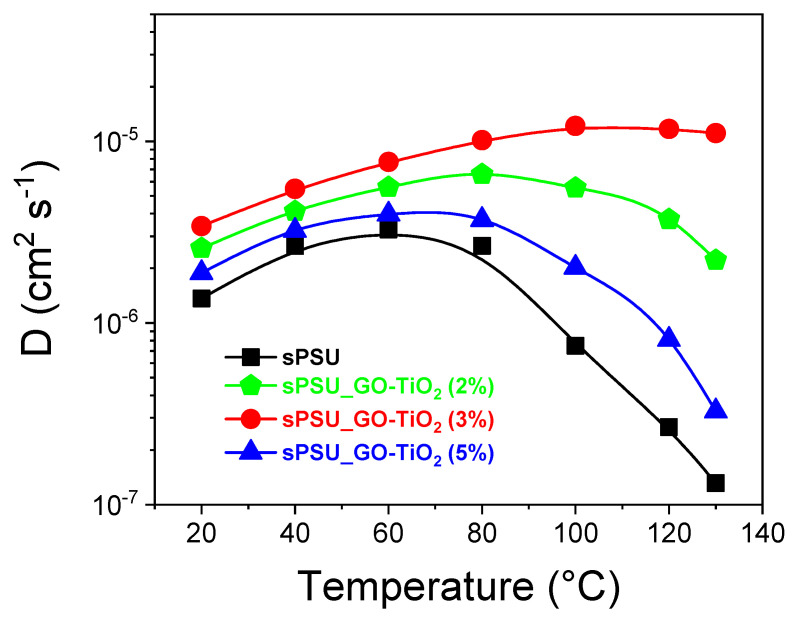
Water self-diffusion coefficient in sPSU and nanocomposite membranes in the temperature range 20–130 °C.

The ^1^H-PFG NMR spectroscopy is a powerful technique to deeply investigate the protons’ transport properties in electrolyte membranes, through the direct measurements of the self-diffusion coefficients (D) [59,60,61,62,63]. Figure 9 shows the temperature behavior, in the range 20–130 °C, of the water diffusivity in the hydrophilic pores of the swollen membranes. D increases with heating due to the thermal energy absorbed by the water molecules, but as it approaches 80–100 °C, the diffusion coefficients start to decrease in value for the nonnegligible evaporation process of the bulk-like water from the membrane (note that during NMR measurements the membrane is not provided with additional humidification). Above this temperature, the contribution to the diffusion coefficient comes from the mobility of hydration water to acid sites of polymer (sulfonic groups) and nanofiller (carboxyl and hydroxyl groups of GO and acid surface of titania). 

However, different behavior was observed among the investigated samples:

(1) Water diffusion is higher in the nanocomposite membranes than in the filler-free sPSU;
(2) The highest values are displayed by the sample at 3 wt.% of loading, which is also the membrane with the highest EIC and WU, confirming that agglomeration of the filler particles and occlusion of the hydrophilic cluster of sPSU occurs at higher loading;
(3) The downfall of D at high temperatures is critical (and similar to the pristine membrane) for the composite sPSU_GO-TiO_2_ 5%, while it is progressively less accentuated in the others, even becoming a plateau for the sPSU_GO-TiO_2_ 3% membrane;
(4) At 130 °C the D value in sPSU_GO-TiO_2_ 3% is almost two order of magnitude higher than in the pristine sPSU, i.e., 1.1 × 10^−5^ and 1.3 × 10^−7^ cm^2^ s^−1^, respectively.

These experimental data highlight, on the one hand, the effectiveness of a hydrophilic hybrid material such as the one proposed, within a polymer matrix, in order to promote both water retention at high temperatures and proton transport properties and, on the other hand, the fact that, only by creating the right polymer-filler network, interconnected routes for the transport of protons in difficult operating conditions can be built.

**Figure 10:**
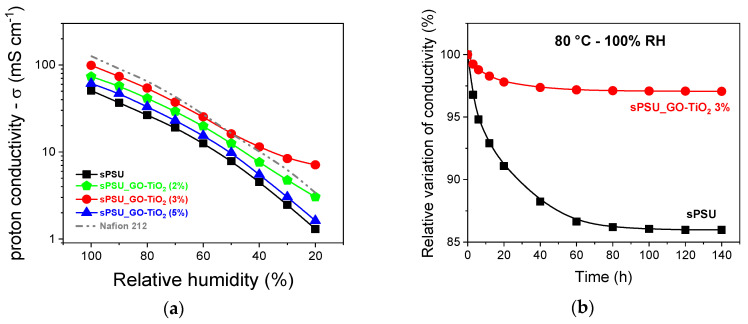
(**a**) Proton conductivity of sPSU-based membranes and commercial Nafion 212 membrane at 80 °C as a function of the relative humidity (RH%), (**b**) relative variation of *σ* in the time (for 140 h), at 80 °C and 100% RH.

Proton conductivity (*σ*) of the membranes at 80 °C and under different RH conditions (from 20 to 100% RH) are shown in Figure 10a in comparison with commercial Nafion 212 membrane as a benchmark. Some representative values are also reported in Table 2. Obtained data are in agreement with the diffusion coefficients seen above.

For pristine sPSU, conductivity at this temperature ranged between about 50 mS cm^−1^ @ 100% RH and 1 mS cm^−1^ @ 20% RH. The best performance was obtained from sPSU_GO-TiO_2_ 3% which, in fully humidified conditions, reached about 100 mS cm^−1^, i.e., twice as much as the sample without filler, but slightly lower than the Nafion benchmark. However, the performance of this hybrid membrane became interesting when the relative humidity was extremely low, which is a prerequisite for the development of PEMFCs able to operate under harsh conditions. In fact, if at RH 50% the proton conductivity is similar to that of Nafion, at RH 20%, it was two-fold higher than the benchmark. This outcome clearly indicates that titanium oxide nanoparticles are able to retain a certain amount of hydration water molecules on their surface and that the hybrid material creates an architecture with the polymer chains, which promote the Grotthuss-type mechanism as a more efficient route for the proton transport [64].

Figure 10b shows the relative variation of *σ* during long-time operation (over 140 h) at 80 °C and 100% RH, for the two representative samples, sPSU and sPSU_GO-TiO_2_ 3%. It was reported that small changes in proton conductivity are due to sufficient hydrolysis stability [65]. In the filler-free sPSU, conductivity reached a plateau after 80 h, with an overall loss of almost 14%, while it was completely negligible in the nanocomposite (less than 3%). The reason for this behavior is related to the partial decomposition that sulfonic acid groups of sulfonated polyaromatic polymers undergoes in aqueous environment. However, the strong electrostatic interaction with the GO-TiO_2_ nanohybrid particles seems to prevent (or strongly delay) the degradation of structural -SO_3_H in sPSU, thus remarkably enhancing the hydrolytic stability of the resulting membrane.

## 4. Conclusions 

TiO_2_ nanoparticles were successfully grown onto graphene oxide platelets by a simple one-pot hydrothermal procedure, and the GO_TiO_2_ nanohybrid material was homogeneously dispersed within a sulfonated polysulfone (sPSU) matrix for the preparation of new nanocomposite electrolyte PEMs, sPSU_GO-TiO_2_, at various filler loadings.

The architecture of the GO-TiO_2_ hybrid material was analyzed by SEM/EDX and well defined by Raman, XRD, and FTIR spectroscopies, obtaining strong evidence that the TiO_2_ nanoparticles are covalently grafted onto the GO platelets. The titania content on graphene platelets was estimated by TGA at about 53% in weight, and SEM images showed that the GO nanoplatelets were completely covered by TiO_2_ nanoparticles, the size of which ranged from 50 to 150 nm diameter. 

The addition of such GO-TiO_2_ hybrid material to the polymer produced a remarkable improvement of the mechanical properties of the nanocomposite membranes, with an increasing of the storage modulus three-fold higher than the filler-free sPSU membrane, as well as a forward shift of the polymer’s glass transition. This is due to the uniform distribution of the filler in the polymer matrix, as evidenced by SEM morphological analysis.

EIS and NMR demonstrated the high water-retention capacity at high temperatures of the composite membrane at 3 wt.% filler loading, as well as a remarkable proton mobility, especially in low relative humidity conditions, suggesting that an architecture between polymer and filler was created with interconnected routes for an efficient proton transport, and marking a step ahead of the state of the art in PEMs.

In addition, the strong interactions between polymer chains and GO_TiO_2_ nanoparticles increased the hydrolytic stability of the composite membrane, since structural degradation phenomena of the sulfonic groups of sPSU were drastically reduced.

## Figures and Tables

**Table 1:** Main chemical–physical characteristic of sPSU-based membranes.

Membrane	IEC [meq g^−1^]	Water Uptake [%]
sPSU	1.36 ± 0.01	27.2 ± 0.1
sPSU_GO-TiO_2_ 2%	1.40 ± 0.02	31.6 ± 0.2
sPSU_GO-TiO_2_ 3%	1.43 ± 0.02	33.4 ± 0.3
sPSU_GO-TiO_2_ 5%	1.42 ± 0.01	31.8 ± 0.2

**Table 2:** Proton conductivity of sPSU and nanocomposite membranes as a function of RH at 80 °C.

Membrane	Proton Conductivity (mS cm^−1^)			
	20% RH	50% RH	80% RH	100% RH
sPSU	1.30	7.84	26.46	50.70
sPSU_GO-TiO_2_ 2%	3.04	12.55	41.39	73.46
sPSU_GO-TiO_2_ 3%	7.11	16.14	54.20	98.91
sPSU_GO-TiO_2_ 5%	1.62	9.81	33.01	61.15
Nafion 212	3.39	16.12	65.90	126.03
